# Complementary and Alternative Medicine Use for Primary Dysmenorrhea among Senior High School Students in the Western Region of Ghana

**DOI:** 10.1155/2019/8059471

**Published:** 2019-11-25

**Authors:** Catherine Samba Conney, Irene Akwo Kretchy, Michelle Asiedu-Danso, Grace Lovia Allotey-Babington

**Affiliations:** ^1^Department of Pharmacy Practice and Clinical Pharmacy, School of Pharmacy University of Ghana, P.O. Box LG 43, Legon, Ghana; ^2^Department of Pharmaceutics and Microbiology, School of Pharmacy University of Ghana, P.O. Box LG 43, Legon, Ghana

## Abstract

**Background:**

Dysmenorrhea is a major gynaecological complaint among females who have reached menarche. It is one of the major causes of absenteeism of females from schools and at the workplaces resulting in loss of productive working hours and work efficiency. Owing to socioeconomic and cultural differences, females from different backgrounds perceive and manage dysmenorrhea differently. Little is known about the use of complementary and alternative medicines (CAM) in the management of this condition by females in senior high schools in Ghana. Thus, this study sought to assess the use of CAM in the management of dysmenorrhea among female students in two senior high schools in Ghana.

**Methods:**

A school-based cross-sectional study using a quantitative approach was conducted on a total of 478 female students attending Archbishop Porter Girl's Secondary School and Mporhor Senior High School. Information on the sociodemographic characteristics, lay representations of dysmenorrhea, pain intensity and severity, quality of life, self-management, and the use of CAM in the management of dysmenorrhea were obtained. The data were analysed using SPSS.

**Results:**

79.3% of the students used some form of CAM to manage dysmenorrhea. Of CAM users, 32% were utilizing mind-body medicine such as endurance and relaxation, 31% used the whole and alternative medicine such as the hot water therapy, 15% used biological-based medicine such as herbal products, and 22% used the manipulative and body-based systems such as exercises. Various CAM methods and products were perceived to be effective in relieving the pain and discomfort associated with dysmenorrhea in about 90% of the participants who used them. Significant associations were reported for pain severity and quality of life (QoL).

**Conclusions:**

This study has demonstrated that the female students experiencing dysmenorrhea employ various CAM remedies in its management. Therefore, there is the need for education on the right management of dysmenorrhea to ensure that safe and efficacious CAM products and methods are used by adolescent female students.

## 1. Introduction

Dysmenorrhea, popularly known as cramps or painful menstruation, is noted as the major gynaecological complaint associated with menstruation worldwide [1]. Dysmenorrhea is a common source of morbidity in both rural and urban populations [2]. The problem is wide spread and its incidence has been reported in Japan (15.8%), India (79.67%), United States of America (85%), Australia (88%), Nigeria (83.1%), Ghana (83.6%), and Ethiopia (85.1%) [3–9].

Some common signs and symptoms associated with dysmenorrhea include lumbago, nausea, vomiting, diarrhoea, and headache [1]. Factors known to be associated with the severity of dysmenorrhea include longer duration menses, younger menarche, obesity, alcohol consumption, cigarette smoking, and there is now even evident that “passive” exposure to tobacco smoke increases susceptibility [10–12].

Dysmenorrhea, however, improves after child-birth [13, 14].

Based on the pathogenesis of the pain, dysmenorrhea can be classified as either primary or secondary. Primary dysmenorrhea occurs as a result of imbalance of prostaglandins in the female reproductive system occurring usually 6–12 months after menarche, while secondary dysmenorrhea is a result of an underlying endometriosis (a condition where the mucous lining that lines the uterus grows outside the uterus), leiomyoma (benign smooth muscle neoplasm in the uterus), adenomyosis (a type of endometriosis where there is a presence of ectopic glandular tissue in the muscles of the uterus), ovarian cysts, and pelvic congestion [1, 15, 16].

Some researchers have argued that females from both the rural and urban settings believe it is not necessary to seek medical attention for dysmenorrhea unless the pain is unbearable and persist for a longer period than it does usually [17]. While insignificant differences exist in the presentation of primary dysmenorrhea by female students of rural and urban dwellings [18], perceptions and management behaviour have, however, been noted to differ [19]. Females in the rural centres have been observed to often exhibit poor reproductive knowledge and are almost always unprepared for menarche. They have been reported to seek information about the problem from their grandmothers, mothers, and friends [20]. These females perceived menstruation as an illness which was shrouded in secrecy, fear, and shame and had devised self-coping strategies and home-based remedies to manage the pain [21]. Unlike rural areas where population is low with limited basic social amenities, urban areas are usually characterized by high human population density and easy access to social amenities. Females in urban areas tend to have difficulties coping with dysmenorrhea and often resort to the use of medications to manage the pain. They reportedly access help from the Internet, community pharmacies, and their teachers where quality heath information about the condition is made available to them [20].

Though both pharmacological and nonpharmacological methods are employed in managing primary dysmenorrhea, the quality of available information may direct the choice of the female to utilize either the pharmacological, nonpharmacological, or both methods. The nonpharmacological therapies may include complementary and alternative medicine (CAM). The use of CAM is wide spread with a progressively increasing utilization over the years in many countries [22]. In Ghana, utilization of CAM has been reported for management and treatment of both chronic and acute disease conditions including hypertension, cancer, malaria, mental disorder, stroke, infertility, and HIV/AIDS, but that for dysmenorrhea is limited [23–27]. With minimal research focusing on the patterns and utilization of CAM in the management of dysmenorrhea, the prevalence and factors associated with CAM by females in rural and urban settings remains to be fully elucidated. When this understanding is increased, it will provide evidence for simple, evidenced-based interventions to be administered to those in secondary schools who experience dysmenorhea. This study was developed to ascertain the prevalence and patterns of CAM use, to report the perceived effect of CAM on dysmenorrhea, and to determine the perceived effect of dysmenorrhea on the quality of life of female students. A better understanding of these associations will direct further research efforts towards optimal reproductive health care using scientifically proven CAM therapies and practices.

## 2. Methods

### 2.1. Study Design

This was a school-based cross-sectional study among female students in two senior high schools in the Western Region of Ghana. Data were collated using a semi-structured questionnaire with both open-ended and close-ended questions.

### 2.2. Study Site

The research was conducted in Archbishop Porter Girls Senior High School (APGSS) and Mporhor Senior High School (MSHS), respectively, located in the urban and rural communities in the Western part of Ghana. It is bordered on the East to the Central Region and on the west to La Cote d'Iviore. It is popularly known for its gold mines and offshore oil platforms. Indigenous people along the coast are mostly fishermen and those landlocked are farmers. The region has 22 districts with an estimated population of 2,376,021 [28].

The APGSS is an all-girls' Catholic educational institution in the Ghana Public Education system in the Sekondi-Takoradi Metropolitan Assembly (STMA). It is specifically located in the administrative capital, Sekondi-Takoradi, which is an urban community. Though it is a catholic institution, girls of all denominations are admitted, and the school provides educational training for over 1,000 females from all over the country. The school runs both boarding and day systems. The school has an infirmary, and approximately 40–60 students report daily with most complaints associated with dysmenorrhea. APGSS was chosen to represent urban participants for the study because most of the students are from the urban centres of the country specifically from Accra, Kumasi, and Takoradi.

The MSHS is a mixed school in the relatively small Mporhor community located in the Mporhor-Wassa East District. The school has a population of over 1,200 students most of whom are from surrounding rural towns Whindo, Adum Bamso, Agona, Benso, and Nsuta. These towns are sparsely populated with very little infrastructure, utilities, and amenities. They all depend on a small district hospital in Mporhor for their health care needs aside the health care centres in each town. The school runs a day and boarding system, but most of the students are day students since the boarding facility can only absorb a handful of students. The school has no infirmary or first aid facility.

### 2.3. Study Participants

Only female students who have reached and experienced menarche and experienced dysmenorrhea within the ages of 13 and 20 were allowed to participate in the study. The exclusion criteria were the female students who do not experience dysmenorrhea or those experiencing dysmenorrhea as a result of an underlying pathophysiology.

The total sample size for study was determined using the statistical formula:

(1) $$\begin{array}{c} n=\frac{t^{2}P\left(1-P\right)}{m^{2}}=293, \end{array}$$

where *n* = required female students, *t* = confidence level at 95% (standard value of 1.96), *P* = estimated prevalence (74.4%) [29], and *m* = margin of error at 5% (standard value of 0.05).

### 2.4. Measures

A 29-item semi-structured questionnaire was employed as the main tool for data collection. Demographic and clinical characteristics of participants such as the age, onset of menstruation, duration of menstruation, the length of cycle, and regularity of cycle were documented. Information on knowledge and lay representation of dysmenorrhea were also obtained. Severity of pain experienced was estimated using the 0–10 Numeric Rating Scale (NRS). The NRS is a unidimensional measure of pain intensity where a respondent selects a whole number (0–10 integers) that best reflects the intensity of their pain [30]. Scores range from 0–10. Higher scores indicate greater pain intensity [31]. The NRS has also been used in Ghana having an inter-rater reliability of 0.93 [32].

The QoL of participants was assessed using the John Flanagan Quality of Life Scale (QoLS). The John Flanagan QoLS is a 15-item instrument that measures five conceptual domains of QoL: material and physical well-being, relationships with other people, social and civic activities, personal development and fulfilment, and recreation [33]. It uses a 7-point scale anchored with the following and scored as “delighted” (7), “pleased” (6), “mostly satisfied” (5), “mixed” (4), “mostly dissatisfied” (3), “unhappy” (2), and “terrible” (1) [33]. The John Flanagan QoLS is scored by adding up the responses on each item to yield a total score of 16–112 where higher scores indicate high QoL. The John Flanagan QoLS has an internal consistency of 0.82–0.92 [34].

Management approaches for dysmenorrhea, covering areas of self-management, cases reported to the hospital, pharmacies and over-the-counter medicine facilities, the use of orthodox medicines, as well as complementary and alternative medicines were also indicated.

### 2.5. Procedure

The data were collected from both schools within two days where a day each was used per school. 250 students were randomly selected per school and gathered in the assembly halls for the data collection exercise. The purpose of the study was explained to them by a member of the research team. The participants filled either the assent or consent forms indicating their willingness to participate in the study. The questionnaires were then distributed, and each question was read out loud by the lead research assistant. Each session lasted for about 2 hours. The participants went through a debriefing exercise upon completion of the questionnaire. They were allowed to talk about their experiences with the data gathering process and to seek clarification on dysmenorrhea.

### 2.6. Ethics

The study was approved by the Institutional Review Board (IRB) of the Noguchi Memorial Institute for Medical Research at the University of Ghana with certified protocol number: NMIMR-IRB 056/15-16. The study protocol and participant consent and assent forms were reviewed and cleared by the IRB.

Approval was also sought from the Ghana Educational Service to allow the chosen schools to participate in the study. Further permission was sought from the administration of each school before data collection. The study was strictly voluntary, and participants consented by signing the consent forms or assent forms for participants below eighteen years.

### 2.7. Data Analysis

The data were sorted, coded, and entered into Statistical Package for the Social Science (SPSS) version 22. Pearson's Chi-squared test was used to test for associations between variables.

## 3. Results

### 3.1. Participant Characteristics

A total of 500 female students approached for the study, and all 500 questionnaires administered were successfully retrieved. However, 22 (4.4%) of the retrieved questionnaires were excluded because the female students did not experience dysmenorrhea, leaving a total of 478 (95.6%) students for the analysis in the distribution of 247 (51.7%) and 231 (48.3%) for the urban and rural schools, respectively. The ages of the female students ranged from 13–20 years with a mean of 17 ± 0.049 years and a median age of 17 years. Most of them reached menarche within the ages of 10–17 years with the mean age of 12 ± 0.069 years. About 55% of them had a 28 days long cycle, 69% had duration of menstruation ranging 2–5 days, and the majority had a very regular cycle. About 47.5%, 33.7%, and 18.8% of them experienced dysmenorrhea on the very first day, after 3 months, and after a year of first menstruation, respectively. In addition to lower abdominal pains, the commonest symptoms of dysmenorrhea were restlessness (55.5%), headache (44.1%), and diarrhoea (26.4%).  Table 1 gives a descriptive statistic of the respondents' characteristics.

### 3.2. Lay Representations of Dysmenorrhea

Of the 478 female students who participated in the study, only 272 (56.9%) participants understood what dysmenorrhea was and could express it in their own words. Of this group, the majority (78%) were from the urban school.  Table 2 shows some lay representations of dysmenorrhea as expressed by some respondents.

### 3.3. Pain Assessment and Quality of Life

The majority of respondents described their pain experience during menstruation as sharp. About 49% of the respondents had pains that lasted two days. Using the pain scales to assess the severity of pain, the majority had moderate pain in both urban (52.6%) and rural (62.3%) schools (Table 3). Students in the urban institution reported higher proportions of severe pain (47.4%) than their counterparts in the rural institution (37.7%). Tables 4 and 5 present results on QoL and the association between pain and QoL (*X*^2^ = 53.3331, *p* value <0.0001 at *p* value ≤0.005), respectively.

### 3.4. Prevalence, Pattern, and Perceived Effectiveness of CAM Use

**Figure 1:**
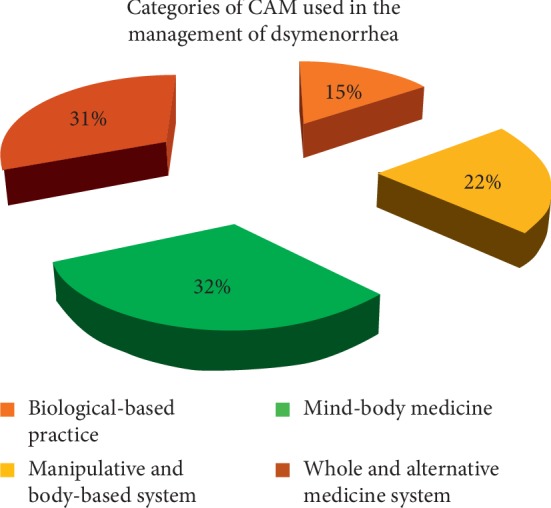
Categories of CAM used.

**Figure 2:**
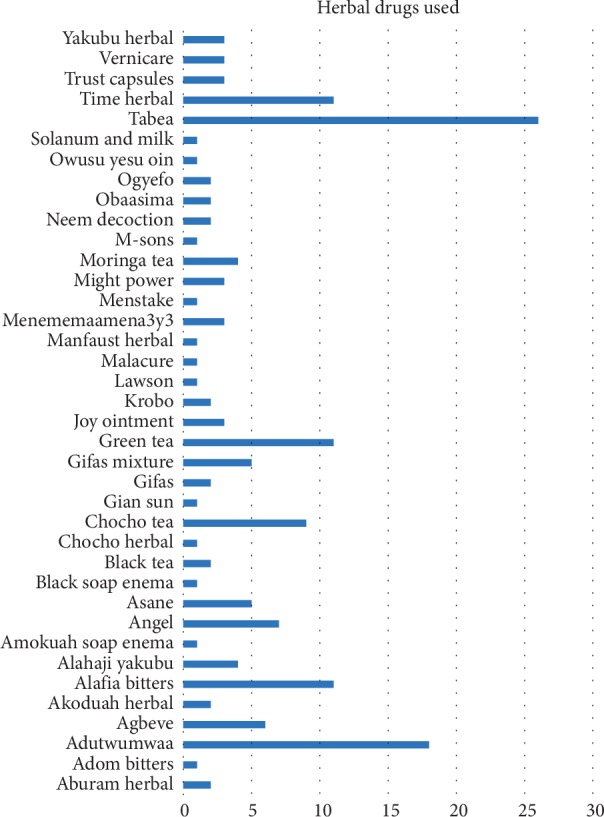
Examples of herbal preparations used by participants.

In all, 79.3% of the students used some form of CAM to manage dysmenorrhea with the majority (53.7%) from the rural institution (Table 6). Of the CAM users, some resorted to herbal medicine while others relied on mind-body medicine and whole and alternative techniques (Figure 1). About 47.9% of the participants who used herbal medicine to manage dysmenorrhea were from the urban institution and 52.1% from the rural school.  Figure 2 shows the types of herbal products the participants used for dysmenorrhea. The various CAM methods and products were perceived to be effective in relieving the pain and discomfort associated with dysmenorrhea in about 90% of the participants.

## 4. Discussion

This study sought to assess the pattern of CAM use in the management of dysmenorrhea among female students in an urban and rural institution. In this study, the mean age for menarche observed was 12 ± 0.069 years similar to what has initially been reported in Ghana and in other countries [29, 35]. However, the female students in the rural institution had a relatively higher mean age compared to those in the urban institution as has been suggested in a previous study that the age of menarche in rural inhabitants are higher than their urban counterparts [36]. The findings of this work suggest that, in all, while about 50% of the female students experienced dysmenorrhea at their first menses, a greater proportion of the students in the rural institution started experiencing dysmenorrhea 3 months after menarche compared to those in the urban setting. It is quite unusual since symptoms of dysmenorrhea tend to be experienced after the first few months of menarche [37]. The need to explore this trend is recommended.

Female students in rural settings have been shown to exhibit poor knowledge in reproductive health and are usually not prepared for menarche and are therefore not abreast with the complications of the reproductive health system [21, 35]. In this study, the majority of female students from the rural institution had poor knowledge about dysmenorrhea. It is thus important to educate these students on the basic information on reproductive health in order to make them know as much about dysmenorrhea as their urban counterparts.

Though dysmenorrhea is not a life-threatening condition on its own, monthly recurrence of severe symptoms represents a significant morbidity with profound negative impact on day-to-day life with compounding emotional distress. While the QoL during dysmenorrhea was comparatively poor among female students in both institutions, those in the urban institution reported more dissatisfaction than those in the rural institution. The perceived reported severity of pain experienced during dysmenorrhea was also associated with QoL. Thus, the more intense the pain is, the greater the dissatisfaction with QoL. This may possibly be attributed to the fact that severe pain affects sleeping patterns, self-esteem, and interpersonal relationships of those who experience the pain [38].

The practice of self-medication appears to be wide spread in the adolescent population with dysmenorrhea. Other studies of dysmenorrhea have also shown that the practice of self-medication and other remedies are common [5, 39]. The most utilized of the five main CAM modalities was the mind-body medicine including relaxation. The relaxation technique has been shown to be an effective method in reducing the symptoms of primary dysmenorrhea in young women [40, 41] and is the most used CAM technique in managing dysmenorrhea [42]. With the whole and alternative medicine, the majority of female students in both institutions used the hot water therapy with the belief that the heat melts the blood, improves the flow, and effectively reduces the pain [43]. Some studies have shown that there is high dependency of rural folks on the use of CAM in the management of several disease conditions including dysmenorrhea [27]. Findings from these studies show that the rural areas are adapting to the situation (dysmenorrhea) by endurance and managing the problem without drugs to a large extent. They utilize self-help techniques such as cold baths, lying supine, heat therapy, and home remedies used in other parts of the world [44, 45]. This study showed no significant association for CAM use in the rural and urban institution. Previous studies have, however, reported high dependency on traditional medicine and herbs for maintenance of basic health care needs by rural folks owing to sociocultural practices, beliefs, and socialization [20, 44].

### 4.1. Limitations

The main limitation of this study is the use of a quantitative design only. Some important perspectives that could have been generated from a qualitative study were not explored. Thus, a mixed methods approach could be adopted in similar subsequent studies. In addition, the perceived effectiveness of the use of CAM in the management of dysmenorrhea was assessed using an item on the questionnaire to elicit a yes or no response. This feedback could be prone to some levels of subjectivity; thus, a more objective measure of effectiveness can be used subsequently. Irrespective of these limitations, the current study has been able to provide some information on the management of dysmenorrhea among adolescent female students in Ghana.

## 5. Conclusion

This study has demonstrated that female students experiencing dysmenorrhea employ various CAM remedies in its management. Therefore, there is the need for education on the right management of dysmenorrhea to ensure that safe and efficacious CAM products and methods are used by the female students.

## Figures and Tables

**Table 1:** Characteristics of participants.

Characteristics		Urban		Rural	
		Frequencies	(%)	Frequencies	(%)
Age(years)	≤15	9	3.6	3	1.3
	16	41	16.6	49	21.2
	17	159	64.4	69	29.9
	18	34	13.8	72	31.2
	19	2	0.8	18	7.8
	≥20	2	0.8	8	3.5
	Mean (17 ± 0.049)				

Onset of menstruation (years)	<10	5	2.0	7	3.0
	10–17	242	98	224	97
	Mean (12 ± 0.069)				

Length of cycle (days)	≤28	50	20.2	36	15.6
	28	132	53.4	131	56.7
	30	65	26.4	64	27.7

Duration of menstruation (days)	≥5	169	68.4	161	69.7
	6–10	78	31.6	70	30.3

Rate of cycle	Very regular	120	48.6	100	43.3
	Somewhat regular	96	38.9	99	42.9
	Irregular	31	12.6	63	27.3

First-time experience of dysmenorrhea	First day	112	45.3	115	49.8
	After 3 months	85	34.4	76	32.9
	More than a year	50	20.2	40	17.3

Signs and symptoms	Headache	119	48.2	92	39.8
	Vomiting	37	15.0	33	14.3
	Fever	29	11.7	27	11.7
	Nausea	53	21.5	46	19.9
	Restlessness	144	58.3	120	51.9
	Backache	45	18.2	15	6.5
	Malaise	14	5.7	17	7.4
	Diarrhoea	85	34.4	45	19.5

Site of pain	Lower back	26	10.5	34	14.7
	Lower abdomen	233	94.3	193	83.5
	Hip	13	5.3	22	9.5
	Thigh	18	7.3	19	8.2

**Table 2:** Lay representations of the definition of dysmenorrhea by some of the female students.

Participant ID	Lay representation
MHSH1	The time of the month where blood comes from the vagina
MSHS2	In my menstrual cycle, sometimes I feel like vomiting when I eat and pains in my abdomen.
MSHS3	Dysmenorrhea is when you have your menses at the age of twelve (12)
MSHS4	I know it is simply menstrual problem
MSHS5	The feeling of restlessness during a girl's menses
MSHS6	It is the feeling of heaviness in the stomach during your period
MSHS7	The serious pain one experiences
MSHS8	When you miss your menses for some time and it starts again
MSHS9	An uncomfortable feeling in the stomach
MSHS10	Some challenges people experience when they menstruate
APGSHS1	I know dysmenorrhea is associated with painful menstruation; it feels like a spring is being pulled from your abdomen.
APGSHS2	The process of having painful cramps during menstruation
APGSHS3	Dysmenorrhea is the burning sensation one feels in the lower abdomen during menstruation.
APGSHS4	It is the tearing of the abdomen with sharp pains
APGSHS5	Pains at the waist side and knee during menstruation
APGSHS6	It is the pain or disorder felt during your menstrual period
APGSHS7	A sharp hurting pain which comes and stops and starts very painful again
APGSHS8	Dysmenorrhea is a condition whereby a female adolescent experiences severe pains at the lower abdomen which is usually called menstrual cramps
APGSHS9	The feeling of being pierced with something in your stomach during menstruation
APGSHS10	Situation where an individual misses her menses

**Table 3:** Details of severity of pain.

	Severity of pain	
	Moderate (1–5)	Severe pain (6–10)
Urban	130 (52.6%)	117 (47.4%)
Rural	144 (62.3%)	87 (37.7%)

**Table 4:** Cross tabulations of quality of life scores.

	Quality of life		
	16–79 (dissatisfied)	80–90 (average satisfaction)	91–112 (satisfaction)
Urban	183 (84.7%)	12 (5.6%)	21 (9.7%)
Rural	116 (69.5%)	20 (12%)	31 (18.5%)

OR: 1.668, 95% CI: 0.929–2.996.

**Table 5:** Association between the severity of pain and quality of life.

	Quality of life		
	16–79 (dissatisfied)	80–90 (average satisfaction)	91–112 (satisfaction)
Moderate pain	165 (70%)	27 (11.4%)	44 (18.6%)
Severe pain	184 (93.4%)	5 (2.5%)	8 (4%)
*X* ^2^ = 53.3331, d*f* = 3, *p* value <0.0001			

**Table 6:** Comparing CAM usage in rural and urban female students.

		Status	
		Urban	Rural
CAM usage	Yes	103	124
	No	144	107
Chi-squared test	*X* ^2^ = 1.034, d*f*. = 1, *p* value = 0.309		

## Data Availability

The data used to support the findings of this study are available from the corresponding author upon request.
